# DNA Methylation Markers from Negative Surgical Margins Can Predict Recurrence of Oral Squamous Cell Carcinoma

**DOI:** 10.3390/cancers13122915

**Published:** 2021-06-11

**Authors:** Bruna Pereira Sorroche, Fazlur Rahman Talukdar, Sheila Coelho Soares Lima, Matias Eliseo Melendez, Ana Carolina de Carvalho, Gisele Caravina de Almeida, Pedro De Marchi, Monique Lopes, Luis Felipe Ribeiro Pinto, André Lopes Carvalho, Zdenko Herceg, Lidia Maria Rebolho Batista Arantes

**Affiliations:** 1 Molecular Oncology Research Center, Barretos Cancer Hospital, Barretos 14784-400, SP, Brazil; brusorroche@hotmail.com (B.P.S.); matias.melendez@professor.fpp.edu.br (M.E.M.); decarvalhoc@iarc.fr (A.C.d.C.); carvalhoa@iarc.fr (A.L.C.); 2 Epigenetics Group, International Agency for Research on Cancer, 69008 Lyon, France; TalukdarF@fellows.iarc.fr; 3 Molecular Carcinogenesis Program, Brazilian National Cancer Institute, Rio de Janeiro 20231-050, RJ, Brazil; sheila.lima@inca.gov.br (S.C.S.L.); monique.lopes@inca.gov.br (M.L.); lfrpinto@inca.gov.br (L.F.R.P.); 4 Pathology Department, Barretos Cancer Hospital, Barretos 14784-400, SP, Brazil; giselecaravina@gmail.com; 5 Medical Oncology Department, Barretos Cancer Hospital, Barretos 14784-400, SP, Brazil; pedro.marchi@medicos.oncoclinicas.com; 6 Oncoclínicas, Rio de Janeiro 22250-905, RJ, Brazil

**Keywords:** DNA methylation markers, margins of excision, oral squamous cell carcinoma, tumor recurrence

## Abstract

**Simple Summary:**

Up to 30% of oral cavity cancer patients with pathologically negative surgical margins usually exhibit tumor recurrence. Early molecular alterations in the tumor-adjacent normal tissues prior to the onset of cancer phenotype could be important to this event. Therefore, here we aimed to evaluate the DNA methylation patterns of negative surgical margins as a prognostic factor for tumor recurrence. Our results demonstrated that recurrent patients with negative histological margins exhibit differential DNA methylation markers. These markers might be crucial for the identification of individuals at higher risk of developing tumor recurrence and could be clinically explored further to help in decreasing morbidity and improving survival rates of these patients.

**Abstract:**

The identification of molecular markers in negative surgical margins of oral squamous cell carcinoma (OSCC) might help in identifying residual molecular aberrations, and potentially improve the prediction of prognosis. We performed an Infinium MethylationEPIC BeadChip array on 32 negative surgical margins stratified based on the status of tumor recurrence in order to identify recurrence-specific aberrant DNA methylation (DNAme) markers. We identified 2512 recurrence-associated Differentially Methylated Positions (DMPs) and 392 Differentially Methylated Regions (DMRs) which were enriched in cell signaling and cancer-related pathways. A set of 14-CpG markers was able to discriminate recurrent and non-recurrent cases with high specificity and sensitivity rates (AUC 0.98, *p* = 3 × 10^−6^; CI: 0.95–1). A risk score based on the 14-CpG marker panel was applied, with cases classified within higher risk scores exhibiting poorer survival. The results were replicated using tumor-adjacent normal HNSCC samples from The Cancer Genome Atlas (TCGA). We identified residual DNAme aberrations in the negative surgical margins of OSCC patients, which could be informative for patient management by improving therapeutic intervention. This study proposes a novel DNAme-based 14-CpG marker panel as a promising predictor for tumor recurrence, which might contribute to improved decision-making for the personalized treatment of OSCC cases.

## 1. Introduction

Head and neck squamous cell carcinoma (HNSCC) is a set of malignant epithelial tumors located in the upper aerodigestive tract [1] and the 7th most frequent cancer worldwide, with 931,931 new cases in 2020 [2]. The oral cavity is the main anatomical site within the head and neck accounting for ~380,000 new cases and ~180,000 deaths in 2020 [2,3]. Oral squamous cell carcinoma (OSCC) is the most prevalent subtype and can originate from the buccal mucosa, the floor of the mouth, tongue, alveolar ridges, palate, and other regions in the oral cavity [3]. OSCC development is strongly associated with lifestyle-related risk factors including smoking and alcohol consumption [4].

OSCC treatment normally involves surgical excision of tumors with adequate surgical margins, followed by radiotherapy and/or chemotherapy [5,6]. Despite the different strategies used and the advances in treatment, the 5-year overall survival rate for OSCC remains around 50% [7,8]. One of the main reasons for treatment failure, together with the depth and pattern of invasion of the tumor, is the development of loco-regional recurrences [9,10], which accounts for approximately 15–35% of the cases [11,12]. Thus, obtaining tumor-free margins at surgery is of paramount importance for decreasing the rates of local recurrence. However, 10–30% of the patients with negative histological margins also exhibit loco-regional recurrences, raising the possibility that the available methods may not be sensitive enough to detect molecular alterations in the tumor surrounding tissue that may precede a histological phenotype [9,13]. The presence of undetected cancerized normal-appearing cells (cells with molecular aberrations) in the surgical margins contributes to increased local recurrence rates and decreased overall survival [14,15,16]. Many studies attempted to identify molecular markers of loco-regional recurrence from pre-operative or post-operative oral brushing samples, but none of them exhibited consistent results with proper specificity and sensitivity of the markers [17,18].

Previous studies have suggested the presence of epigenetic alterations in the mucosa adjacent to the primary tumor, before the onset of cancer phenotype, due to which it is usually not visible by pathological examination [13,14,19,20]. However, most of these studies were limited to analyzing the promoter regions of genes related to carcinogenesis. Hence, the comprehensive genome-wide analyses of DNA methylation (DNAme) profile of negative surgical margins of OSCC might be able to contribute to a better understanding of the recurrence patterns of these patients.

Therefore, we hypothesized that pathologically confirmed negative surgical margins of OSCC cases may harbor aberrant DNAme that could be explored as potential targets to predict tumor recurrence. To test this hypothesis, we performed DNA methylome analysis on negative margin tissues from recurrent and non-recurrent OSCC cases. We then replicated our results using normal adjacent to tumor (NAT) samples of HNSCC cases from The Cancer Genome Atlas (TCGA) database. We found a set of 14 CpGs that could be used to differentiate patients with a higher risk of developing local recurrences. This marker panel could help to improve the prediction of prognosis in OSCC cases.

## 2. Materials and Methods

### 2.1. Patients

The discovery phase of this study included 32 patients surgically treated for primary OSCC at the Department of Head and Neck Surgery of the Barretos Cancer Hospital (BCH), Brazil, from 2007 to 2015. All patients had complete clinicopathological data available, including local recurrence status. Tumor staging was determined based on the 7th edition of the American Joint Committee on Cancer (AJCC) TNM classification system. Previously untreated patients with primary OSCC submitted to surgery with curative intent and confirmed negative surgical margin samples were the inclusion criteria. Patients were excluded if they underwent neoadjuvant treatment or presented synchronous tumors. In addition, patients submitted to an adjuvant treatment or with less than two years of follow-up that did not develop local recurrence were excluded. The study was approved by the BCH Research Ethics Committee under approval number 1121/2016.

### 2.2. Sample Preparation for Methylome Profiling

**Figure 1:**
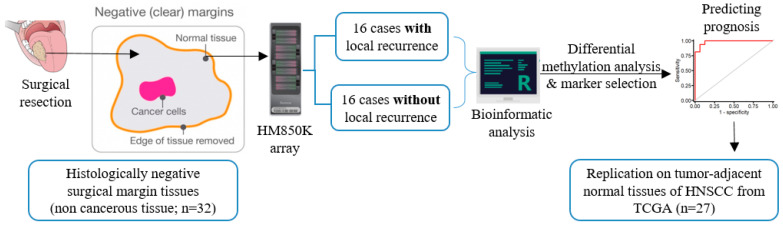
Study design showing sample sources and processing scheme for DNAme analysis and identification of markers.

The biospecimens analyzed in this study consisted of fresh-frozen histologically negative surgical margins tissue from OSCC patients. At BCH, after OSCC excision, the surgical specimen is routinely sent for anatomopathological evaluation of the tumor tissue and surgical margins through the preparation of paraffin blocks and hematoxylin and eosin (H&E) slides. For the purpose of this study, a fragment of the mucosal margin was also sent for cryopreservation at the Biobank of the institution. According to the availability of stored material, one cryopreserved mucosal margin fragment from each patient was used. A new H&E slide prepared from the frozen mucosal margin was evaluated by an experienced pathologist for confirmation and characterization of cellular components prior to DNA isolation (Supplementary Figure S1). The average distance of the closest negative margins from the tumor area was 6 mm. DNA extraction was automatically performed in the QIAsymphony SP equipment (Qiagen, Hilden, Germany), following the manufacturer’s instructions. Quantification of the samples was performed by fluorimetric assay, using the Qubit dsDNA BR Assay Kit and Qubit Fluorometer 2.0 equipment (Invitrogen, Carlsbad, CA, USA). The purity of the DNA was determined by optical density in NanoDrop 2000 (Thermo Fisher Scientific, Waltham, MA, USA). Samples with insufficient quantification or quality were replaced. Then, 600 ng of DNA was bisulfite converted using the EZ DNA Methylation Kit (Zymo Research, Irvine, CA, USA), according to the manufacturer’s protocol [21]. The study design demonstrating the sample processing plan for DNAme analysis is shown in Figure 1.

### 2.3. DNA Methylome Profiling

Bisulfite-converted samples were subjected to genome-wide DNAme profiling using the Illumina MethylationEPIC BeadChip microarray (HM850K; Illumina, San Diego, CA, USA) [21]. This array provides wide coverage across the genome, interrogating more than 850,000 CpG sites and allowing a comprehensive view of genome-wide DNAme patterns of various genomic regions [22]. The array generates intensity data (IDAT) files containing the raw intensity data values for every bead consisting of CpG probes defining the DNAme status of every interrogated CpG site.

### 2.4. Data Pre-Processing and Normalization

The IDAT files generated from the HM850K arrays were used for the analysis. The pre-processing and normalization were done using the “Funnorm” function present in minfi Bioconductor package in R (version 3.6.3) [23]. This eliminates undesirable discrepancy by regressing out variability explained by the control probes present on the HM850K array. The CpGs that showed low detection *p*-value or low confidence (detection *p* > 0.05) or missing data for >10% of samples were excluded. Moreover, single nucleotide polymorphisms (SNPs) associated with CpG probes [24], cross-reactive probes [22] and probes from X and Y chromosomes were excluded. Samples with overall low confidence for >10% of CpG sites or missing/poor signals were also removed from the analysis. After the pre-processing, normalization, and probe filtering, 706,671 probes within the 32-margin samples were retained for further analysis. Intensity data for each CpG site were assigned with a β-value [25], calculated by the ratio of signal intensities of methylated probe (Mp) versus the sum of Mp and unmethylated probe (Up):

(1) $$\beta \;=\frac{\mathrm{Mp}}{\left(\mathrm{Mp}\;+\;\mathrm{Up}\right)}$$

This value ranges from zero to one, with one being fully methylated and zero completely unmethylated. For further analyses, β-values were also converted to M-values that is calculated as the log2 ratio between the intensities of Mp and Up [26]:

(2) $$M\;=\log_{2}\left(\frac{\mathrm{Mp}}{\mathrm{Up}}\right)$$

Principal Component Analysis (PCA) was performed to identify the influence of different variables in various components. Potential unknown sources of variation were adjusted by the Surrogate Variable Analysis (SVA) R Bioconductor package [27] and additional known variables were adjusted during the regression analysis. The bioinformatics analysis was performed following the pipeline available in https://github.com/IARCbioinfo/methylkey (accessed on 30 April 2020).

### 2.5. Differential DNAme Analysis

We performed robust linear regression analysis using the Limma package [28] to identify differential DNAme between recurrent and non-recurrent samples. To identify Differentially Methylated Positions (DMPs), we corrected for multiple testing with False Discovery Rate (FDR) < 10% [29] and set the cut-off of at least 5% Δβ (≥5% difference in DNAme). Since we had a limited sample size and included histologically normal squamous epithelial samples in our study, we considered less stringent thresholds in our analysis.

A pathway enrichment analysis using all DMP-related genes was performed using the Kyoto Encyclopedia of Genes and Genomes (KEGG) database using the Enrichr tool [30,31]. Enrichr tool uses a computational method for deducing knowledge about an input set of genes (DMP associated genes in our case) through comparison to annotated sets of genes corresponding to various pathways that represent prior biological knowledge. The generated *p*-values are calculated by Fisher’s exact test, with genes being considered independent, while the q-values represent adjusted *p*-values calculated using the Benjamini–Hochberg method [32].

To identify Differentially Methylated Regions (DMRs) consisting of ≥2 CpGs within the 1000 base-pair window, we used the DMRcate package with FDR of <5% [33]. The DMR analysis allows the identification of regional differences between the groups, improving the robustness of the findings and the replicative value when compared with individual site differences [34]. To estimate and illustrate region-based associations, we used Gviz package [35].

Annotation of the CpG probes was done using specific Bioconductor packages for HM850K array and HM450K array data [36,37]. ChIPseeker package was used to illustrate DMPs and DMRs genomic localization [38]. The comparison between two observed proportions was assessed using the two-proportion z-test.

### 2.6. TCGA Replication

To replicate our results from the discovery phase, we downloaded Illumina HumanMethylationBeadChip array (HM450K) raw data of HNSCC-NAT samples (IDAT files) from TCGA using TCGAbiolinks package [39]. In addition to the HM450K data, clinical and demographic data were also retrieved from cBio Cancer Genomics Portal [40]. From the TCGA downloaded methylome data, 27 NAT samples from HNSCC were included in our study, after excluding samples from patients who underwent neoadjuvant treatment, who presented synchronous tumors or were in the disease-free group for less than one year of follow-up. These HM450K data were stratified based on recurrence-free survival (RFS), with 19/27 showing RFS and 8/27 showing recurrence in five years. RFS was defined as the time between surgery and the advent of a new tumor event. The differential DNAme analysis between these groups was conducted following the same pipeline as the discovery set, by performing a linear regression based on the RFS status of the patients to identify DMPs and DMRs.

It is important to mention that the NAT samples from TCGA differ in their proximity to the tumor sites when compared to the surgical margin samples included in the discovery set, which could lead to a different field cancerization effect. However, our study hypothesizes that the pathologically confirmed normal adjacent tissue—regardless of the distance to the tumor—might harbor altered DNAme that could be explored as potential targets to predict tumor recurrence.

### 2.7. Assessment of Recurrence Markers

To assess and identify a set of CpGs that could discriminate recurrent cases from non-recurrent ones, we performed Partial Least Square-Discriminant Analysis (PLS-DA) using β-values of the identified DMPs [41]. PLS is a supervised analysis that uses the independent βvalues from DNAme to predict the dependent variables as the outcome (recurrence). Here, we determined the potential CpG markers from the previously identified common DMPs in the discovery and TCGA set, based on the least error rate and the lowest number of predictors (CpGs), possible using cross-validating with 100 iterations [42]. We also estimated the performance of the identified CpG sets by plotting receiver operating characteristic curves (ROC) using discovery set samples and then the performance was validated using TCGA samples. Once the CpG markers were selected, a linear regression was performed to assess the relationship between the methylation pattern of these markers and the recurrence status of the patients. Then, a risk score for each patient was calculated by summing up the multiplication of the β-values of each CpG with its respective regression coefficient, as described below:

(3) Risk Score= β1∗ coef1+ β2 ∗coef2+…+ βn∗coefn

In order to assess the influence of field of cancerization and tumor anatomical sites in the identified DNAme patterns, β-values from 144 TCGA tumor samples were downloaded from the LinkedOmics database [43]. Similar to our discovery set samples, for this analysis, disease free survival data were obtained from patients with negative surgical margins and with tumors of the tongue, floor of mouth, or alveolar ridge. Methylation values of the target CpG sites were compared between anatomical sites and recurrence groups.

### 2.8. Survival Analysis

The methylome analysis results were correlated with the clinical and pathological data of the patients, especially with local recurrences. The cases from the discovery and TCGA set were stratified by the median value of the developed risk score. The differences in each set of data between the two groups were evaluated using the Student *t*-test. The RFS analysis of the stratified groups was performed by the Kaplan–Meier method and the survival curves were compared using the log-rank test (R package survminer) [44] with a statistical significance of *p*-value ≤ 0.05.

## 3. Results

### 3.1. Clinicopathological Characteristics

This study consists of 32 OSCC cases from the Barretos Cancer Hospital patient cohort. Half of the subjects (*n* = 16) developed local recurrence within five years after initial treatment. The average age of the cases was around 60 years, with 84.4% reporting tobacco and 71.9% alcohol consumption. Most of the patients were male (68.8%). The main anatomical site affected in the cases was the tongue (18/32; 56.3%) followed by the floor of the mouth (11/32; 34.4%). The clinicopathological characteristics of the cases included in the discovery phase are shown in Table 1.

In the TCGA cohort, eight patients were described as recurred or progressed, whereas 19 patients were free of disease at the time of follow up. The clinicopathological data of this cohort are indicated in Table 1. The average age of the patients was 60 years. Most of the patients were male (81.5%) and tobacco (76.9%) and alcohol (76.9%) consumers. The main anatomical sites were the tongue (48.2%) and the larynx (37.0%), and most of the patients were diagnosed with advanced tumors (92.6%).

### 3.2. Identifying Recurrence-Associated DMPs

**Figure 2:**
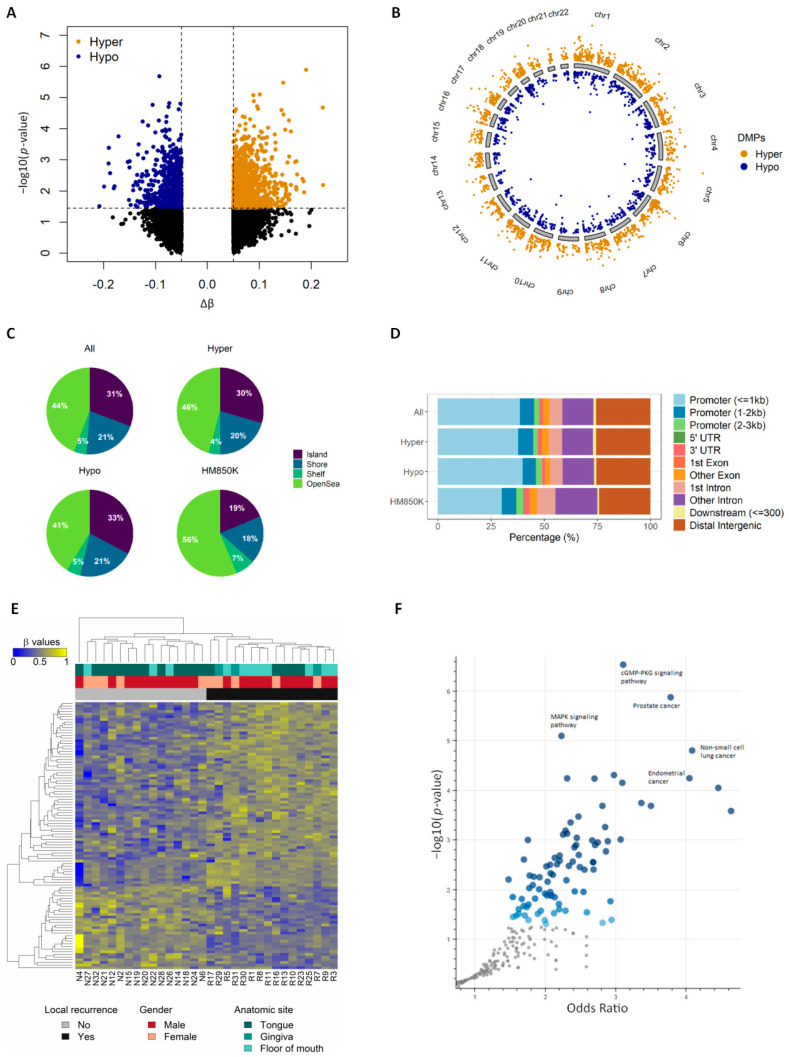
Identifying tumor-specific DMPs (5% Δβ) across the genome. (**A**) volcano plot showing hyper and hypo methylated CpGs between recurrent and non-recurrent cases. The *x*-axis representing the differential methylation between the groups is plotted against the statistical significance of each probe analyzed. Hypermethylated probes with adjusted *p*-value > 0.1 and Δβ ≥ 0.5 are represented in orange, while hypomethylated probes with adjusted *p*-value > 0.1 and Δβ ≤ −0.5 are represented in blue; (**B**) genome-wide DNA methylation profile of patients that developed local recurrence, by chromosome. Orange dots represent hypermethylated probes, blue dots represent hypomethylated probes; (**C**) CpG context of the identified DMPs in CpG islands, shores, shelves, and open sea. All (*n* = 2512), hypermethylated (*n* = 1594) and hypomethylated (*n* = 918) DMPs are compared with the HM850K array (*n* ≅ 85,000); (**D**) genomic annotations of the identified DMPs in the human genome. Plot generated using ChIPseeker package; (**E**) heatmap showing the top 100 DMPs associated with local recurrence. CpG sites are clustered in the horizontal rows and samples are clustered in the vertical columns. High methylation levels are shown in yellow and low methylation levels in blue, according to the scale bar on the left; (**F**) volcano plot showing the significance of each gene set from the DMP-associated gene list versus its odds ratio. Each point represents a single gene set from a pathway, where the larger and darker blue color dots represent significant (*p*-value < 0.05) and smaller gray color dots represent non-significant enrichments.

The quality of HM850K array-generated IDAT files from all samples was considered good, as they exhibited small detection *p*-values, indicating the reliability of the signal [45] (Supplementary Figure S2A). Since age, gender, and alcohol consumption were found to be important variables based on principal component analysis (PCA), we adjusted these variables in the regression model. Differential methylation analysis revealed 2512 DMPs associated with tumor recurrence (Supplementary Table S1) with 5% Δβ difference and FDR of 10% (genomic inflation factor λ = 0.99; Supplementary Figure S2B). The majority of DMPs (63.5% i.e., 1594/2512) were hypermethylated in the recurrent cases (Figure 2A). The median distribution of hypermethylated probes throughout the chromosomes was 64.3% (50–70.9%), whereas the median distribution of hypomethylated probes was 35.7% (29.1–50%; Figure 2B). The identified DMPs showed enrichment in regulatory regions such as CpG islands (31% vs. 19%, *p* < 0.001) and shores (21% vs. 18%, *p* < 0.001) compared to the total number of probes present in the HM850K array (Figure 2C). Conversely, there was a decrease in the proportion of DMPs in open sea regions, when compared with HM850K (44% vs. 56%, *p* < 0.001; Figure 2C). Genomic localization of the identified DMPs revealed that they are largely in the ≤1 kb promoter regions (38.5% vs. 30% in the entire array), corroborating with the previous result and suggesting their potential role in altering gene expression (Figure 2D). No significant differences were observed when comparing genomic localization within hypermethylated and hypomethylated DMPs. We prioritized the 100 most significant DMPs found when comparing samples with and without recurrence. As expected, the use of those DMPs in an unsupervised hierarchical clustering separated the cases according to their recurrence status (Figure 2E). Recurrence status of the cases were the major clusters and the clustering pattern was not influenced by gender and tumor anatomical sites.

We used the CancerMine resource [46] to retrieve information about the role of the 100 DMP-related genes as oncogenes and/or tumor suppressor genes (TSG) in cancer. Among these, twenty-six genes (26/100, Figure 2E) had previously been reported to have a role in cancer: 30.8% (8/26) were TSG, 50% (13/26) were oncogenes and the remaining five (19.2%) had conflicting associations. Only one DMP-related gene (*NSD2*) was described as associated with HNSCC.

To get insights into the biological impact of altered DNAme patterns associated with recurrence, a pathway enrichment analysis using all 1508 unique DMP-related genes (Supplementary Table S1) was performed. cGMP-PKG signaling, MAPK signaling, prostate, lung, and endometrial cancers were among the top enriched and significant pathways identified (Figure 2F and Supplementary Table S2). This signifies the potential DNAme-associated with the deregulation of cell signaling and cancer-related genes and pathways.

### 3.3. Identifying Recurrence-Associated DMRs

**Figure 3:**
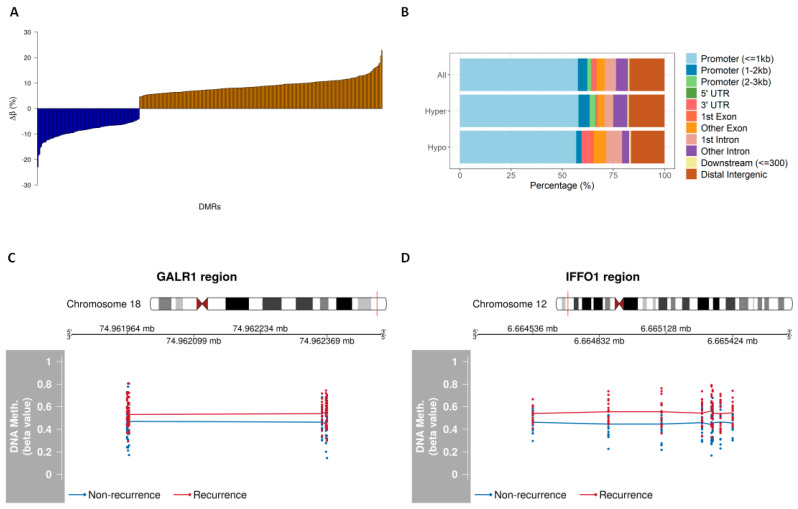
Genomic characterization of DMRs (5% Δβ). (**A**) Distribution of the percentage of differential DNAme of the 392 identified DMRs between recurrent and non-recurrent groups; (**B**) genomic annotations of the identified DMRs in the human genome. Plot generated using ChIPseeker package; (**C**) DMR plots showing chromosome localization and the differential DNAme of *GALR1* and (**D**) *IFFO1* regions. The average methylation values in recurrent (red) or non-recurrent (blue) cases are demonstrated.

DMR analysis by considering at least two or more differentially methylated CpGs in a 1 kb window revealed 392 regions with different DNAme patterns between recurrent and non-recurrent groups. Among the identified DMRs, 276/392 (70%) were hypermethylated and 116 (30%) hypomethylated in the recurrent group with a mean Δβ of 5% (Figure 3A and Supplementary Table S3). The majority of these DMRs were located within CpG islands, which are usually present in the promoter regions. The genomic characterization of these DMRs revealed more hypomethylation of the recurrent group in the first intron (*p* = 0.13), other exons (*p* = 0.16), and 3′ untranslated (UTR) regions (*p* = 0.02). We also found more hypermethylated DMRs of the recurrent group in the promoter (0.07) and other introns (*p* = 0.14; Figure 3B). Among the top DMRs were *GALR1* and *IFFO1*; the DMR at *GALR1* constitutes six CpGs and the DMR at *IFFO1* is comprised of eight CpGs. All CpGs within these regions were hypermethylated in recurrent cases (Figure 3C,D).

### 3.4. Identification of Potential Markers of OSCC Recurrence

**Figure 4:**
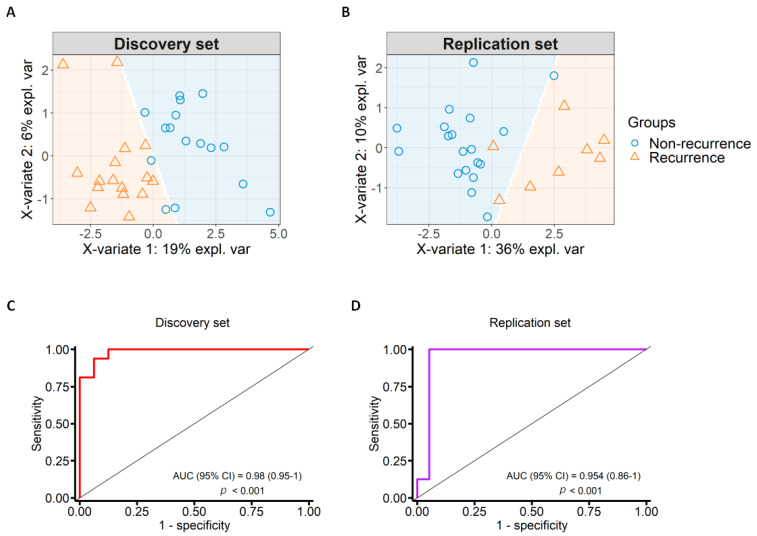
Marker selection with specificity and sensitivity test. (**A**) Sample prediction area from a PLS-DA model based on the 14-CpG marker panel from the discovery set; (**B**) sample prediction area from a PLS-DA model based on the 14-CpG marker panel in the replication set from TCGA; (**C**) Receiver Operating Characteristic (ROC) curve showing specificity and sensitivity of the identified DNAme markers in the discovery set. The area under the curve (AUC) was 0.98; (**D**) ROC curve showing specificity and sensitivity of the identified DNAme markers in the replication set from TCGA. The AUC was 0.95.

PLS-DA identified 14 CpG sites associated with OSCC recurrence that discriminate samples into recurrent and non-recurrent groups in our discovery set analysis by cross-validating with 100 iterations (Figure 4A and Table 2). These identified 14 CpGs also correctly predicted the recurrence status in samples from TCGA (Figure 4B). We performed multidimensional scaling (MDS) plots using the 14-CpG marker panel stratifying the patients based on risk factors and other clinical characteristics to evaluate the influence of these parameters on DNAme patterns (Supplementary Figure S3). We observed that samples were dispersed randomly after stratifying by various parameters suggesting minimal or no influence of these in DNAme patterns. Furthermore, the methylation of the proposed 14-CpG panel was plotted in box-plots and samples were stratified based on various clinical parameters (Supplementary Figure S4). We found statistically significant differences in DNAme based on recurrence even after stratifying by these parameters.

Moreover, we tested the possibility of any bias due to tumor anatomical sites in the identified DNAme patterns by MDS plot using the 14-CpG panel and then tested if this was also true for TCGA tumor tissues. We did not see any significant bias and these results further substantiate that the identified recurrence-specific DNAme patterns are not influenced by different tumor anatomical sites in our discovery set (Supplementary Figure S5A) and TCGA tumor samples (Supplementary Figure S5B).

The sensitivity and specificity of the identified marker panel in predicting recurrence were assessed using the Receiver Operating Characteristic Curve (ROC) analysis to evaluate the predictive accuracy in the discovery and TCGA datasets. The Area Under Curve (AUC) of the identified 14 CpG markers for the discovery set was 0.98 (*p* = 3 × 10^−6^; CI: 0.95–1) and the replication set (from TCGA) was 0.95 (*p* = 2 × 10^−4^; CI: 0.86–1) shown in Figure 4C,D.

**Figure 5:**
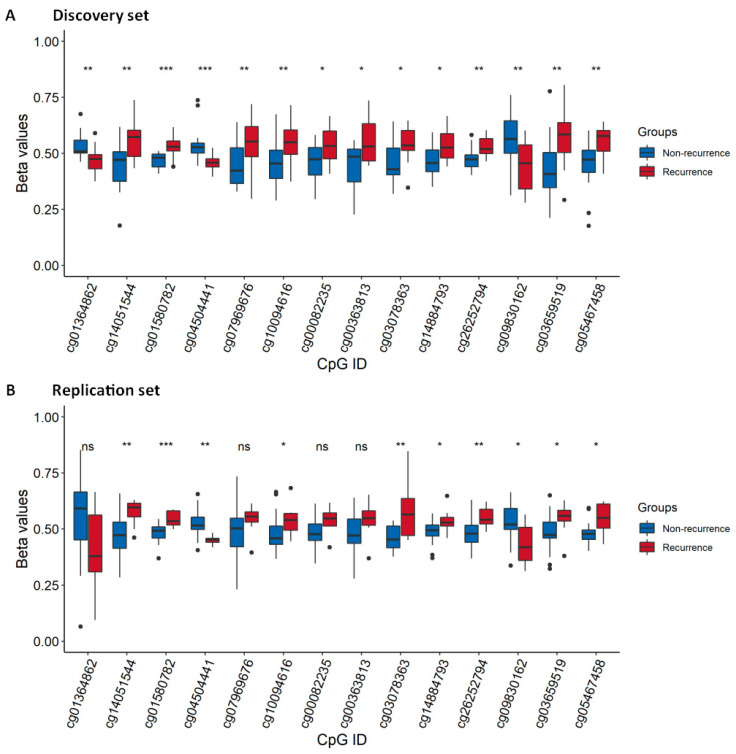
Box-plots comparing the β-values of the identified 14-CpG marker panel between recurrent and non-recurrent groups. (**A**) Discovery and (**B**) replication sets stratified by recurrence status. Middle line in each box represents the median; lower and upper box show the first and third quartiles, respectively. Whiskers represent the 95% confidence interval of the median. *** *p* < 0.001; ** *p* < 0.01; * *p* < 0.05; ns: not significant.

The DNAme status of the identified 14-CpG panel was estimated in the discovery and TCGA set of samples. The β-values of recurrent and non-recurrent cases were significantly different for almost all the 14 CpGs in both the discovery (Figure 5A) and TCGA sets (Figure 5B). Among the TCGA sample set, four CpGs namely: cg01364862, cg07969676, cg00082235, and cg00363813 were not significantly different; however, they showed a similar trend of alteration as the discovery set.

We also evaluated the DNAme of 7 common CpGs from the 14-CpG marker panel in 144 TCGA tumor tissues. Among the cases, 85 remained disease free and 59 exhibited recurrence/progression. The mean methylation of the common CpGs was significantly higher (*p* = 0.04) in the recurrence group.

### 3.5. Survival Analysis Using Combined Score Generated from the Identified Marker Panel

**Figure 6:**
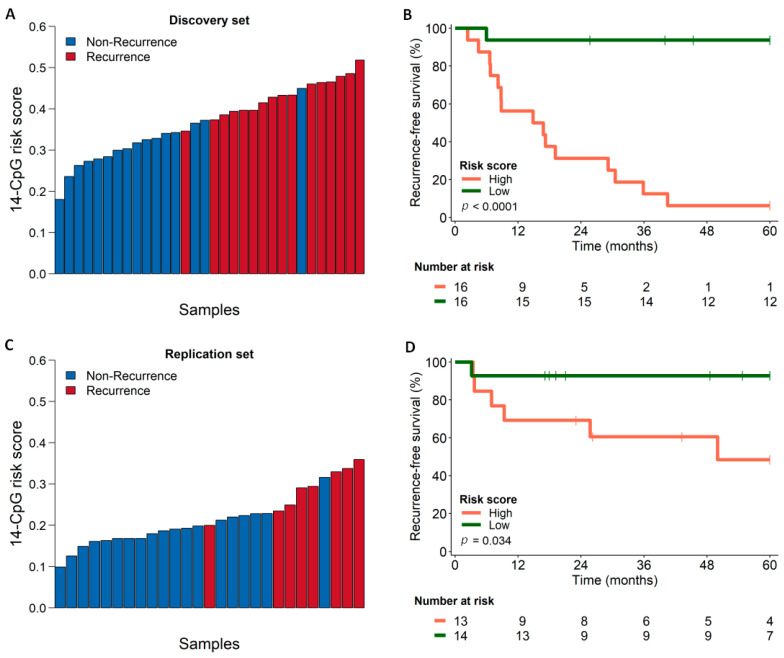
Scoring and survival curves. (**A**) Calculated score for each of the cases from the discovery set using the identified 14-CpG marker; (**B**) Kaplan–Meier curves showing the recurrence-free survival patterns of the cases from the discovery set, comparing cases with low (green line) and high (orange line) risk of developing recurrences; (**C**) scoring for each case from TCGA replication set using the identified 14-CpG marker; (**D**) Kaplan–Meier curves showing the disease-free survival patterns of the cases from the replication set, comparing cases with low (green line) and high (orange line) risk of developing recurrences.

Based on the DNAme status and the regression coefficient of the identified 14-CpG marker panel, a risk score was generated to stratify the cases for predicting their recurrence. We stratified the cases into two groups based on the median value of the generated risk score. The risk scores of the discovery set are shown in Figure 6A, which ranged from 0.1805 to 0.5186 with a median value of 0.3730. The cases with values above the median were considered in the group of high-risk scores and the cases with scores lower than the median were considered in the low-risk score group. Kaplan–Meier curve plotted for recurrence-free survival between the two groups of the discovery set showed better survival for the low-risk score individuals than those with high-risk scores (*p* < 0.0001), presented in Figure 6B. The cases from the TCGA set were also stratified similarly based on the risk scores which ranged from 0.0991 to 0.3593 with a median value of 0.1996 (Figure 6C). Kaplan–Meier curve plotted for recurrence-free survival between the two groups of the TCGA set also revealed better survival for the low-risk score individuals than the high-risk scores (*p* < 0.034) shown in Figure 6D. Half of the individuals from the high-risk group exhibited recurrence within five years, which further justifies the validity of the scoring and the identified marker panel.

## 4. Discussion

Surgical resection is the most crucial step in the treatment of OSCC which includes resection of negative margins to assess the effectiveness of the surgical procedure. However, up to 30% of patients who present histologically negative surgical margins at the time of surgery will develop recurrences [14]. This is probably due to the residual cluster of tumor cells in the negative margins that are undetectable upon routine histopathological examination (minimal residual cancer) [47]. Another possible theory suggests the presence of atypical changes in the tissues surrounding the tumor and associates their persistence with the development of local recurrence or second primary tumors, which is also called the theory of field cancerization [48]. Both concepts are closely related to an insufficient histological evaluation of apparently normal tissues surrounding the tumor, suggesting that additional methods should be explored for greater accuracy in the evaluation of surgical margins. Therefore, it was proposed that the cells from negative margins might harbor additional genetic and epigenetic alterations leading to invasive cancer at a later stage [48,49].

Most of the previously reported studies focused on specific DNAme events with a targeted approach in head and neck cancers [50,51,52,53,54,55]. This is the first study to investigate the recurrence-associated genome-wide DNAme alterations in negative surgical margins of OSCC to predict local recurrence. Our study revealed recurrence-specific aberrant DMPs and DMRs and the identified DMP-associated genes were enriched in cell signaling and cancer-related pathways. This further provides additional evidence for the presence of DNAme alterations of key cancer-related genes and pathways in negative surgical margins. The top differentially methylated probes could distinguish cases based on recurrence status, irrespective of other variables like gender or anatomical site, which establishes the robust nature of the identified aberrant DNAme patterns.

We also proposed a DNAme-based 14-CpG marker panel for predicting OSCC-recurrence. The identified 14 CpG marker panel had a good performance in discriminating the recurrence status of discovery set cases and also cases from TCGA. Among the genes associated with the 14-CpGs in the proposed marker panel (Table 2), three genes were earlier associated with HNSCC carcinogenesis. Promoter hypermethylation of the *GALR1* gene was associated with a decrease in disease-free survival of head and neck cancer patients and its potential use as a biomarker for prognosis was suggested [56,57]. The lack of *SOX7* expression was associated with advanced tumor stages, regional lymph node metastasis, and worse prognosis in OSCC patients [58]. Furthermore, the methylation of a CpG panel including *HOXC4* was predictive of survival in patients with oral cavity cancers [59]. In addition to these, we also identified some novel genes among the 14-CpG markers that were not previously associated with cancer recurrences such as *IFFO1*, *ALKAL1*, *FAHD1*, *FSTL4*, *SLC7A9*, *IQCJ-SCHIP1, CLDN11* and three other CpGs at intergenic regions.

These findings could be useful for predicting OSCC recurrence that could benefit from additional therapies or new targeted therapeutic trials due to the presence of cells with molecular alterations in the histologically normal surrounding tissues. Moreover, these results support the notion of reassessing the routine surgical margins pathological evaluation process at the molecular level to improve the prognosis of OSCC patients. This could be done by coupling the molecular analysis (targeted DNAme analysis) of the surgical negative margins in addition to the histopathological analysis, which is routinely practiced.

Our results not only suggest the effectiveness of the identified recurrence-specific DNAme events for the OSCC prognosis but also establish the presence of early epigenetic alterations in the negative surgical margins that cannot be detected by routine histopathology. Among the limitations of our study is the small sample size. This is partly because of the low yield of DNA recovered from non-tumor tissues [60,61], whereas a relatively high-quality DNA sample is required for performing the HM850K array. We have overcome this limitation to some extent by replicating the results on an independent set of samples using TCGA data. However, we included other anatomical sites from head and neck cancers beyond the oral cavity in the replication set, owing to the lack of prognostic information available for all OSCC cases from TCGA and the limited number of tumor-adjacent normal tissue DNAme data availability. Additional studies with a larger sample size are required to further validate the robustness of the identified marker panels. Overall, our identified marker panel exhibited promising results using DNA from post-operative negative margin tissues and might be further tested on oral exfoliated cells (even by pre-operative sampling) to validate its efficacy and clinical significance.

## 5. Conclusions

In conclusion, our study confirmed that there are DNAme alterations in the histologically normal mucosa adjacent to the primary tumor of OSCC cases. These alterations might act as early driver events for local recurrences in these individuals. Moreover, these DNAme patterns can serve as markers to differentiate patients with a higher risk of developing local recurrences and predicting prognosis.

## Figures and Tables

**Table 1:** Clinicopathological characteristics of the patients.

Characteristic	Discovery Set *n* (%)	Replication Set *n* (%)
Mean age (range)	62 (45–89)	59.6 (26–80)
Gender		
Male	22 (68.8)	22 (81.5)
Female	10 (31.3)	5 (18.5)
Tobacco Consumption		
Never	5 (15.6)	6 (23.1)
Ever	27 (84.4)	20 (76.9)
Alcohol Consumption		
Never	9 (28.1)	6 (23.1)
Ever	23 (71.9)	20 (76.9)
Anatomical Site		
Floor of mouth	11 (34.4)	2 (7.4)
Tongue	18 (56.3)	13 (48.2)
Gingiva	3 (9.4)	1 (3.7)
Larynx	0 (0.0)	10 (37.0)
Base of tongue	0 (0.0)	1 (3.7)
Tumor Stage		
I/II	19 (59.4)	2 (7.4)
III/IV	13 (40.6)	25 (92.6)

**Table 2:** Information related to the 14 CpGs in the marker panel.

CpG-ID	Chr	Start	End	RefGene	Genomic Location	*p*-Value	Average β-Values		Δβ
							R	NR	
cg09830162	16	1,889,615	1,889,614	*FAHD1*	3′UTR	<0.001	0.437	0.562	−0.125
cg04504441	5	132,948,218	132,948,217	*FSTL4*	1st exon	<0.001	0.459	0.540	−0.081
cg05467458	19	33,361,033	33,361,032	*SLC7A9*	TSS1500	0.002	0.555	0.450	0.106
cg07969676	8	10,590,642	10,590,641	*SOX7*	5′UTR	0.002	0.548	0.451	0.097
cg03078363	12	54,408,665	54,408,664	*HOXC4*	TSS1500	0.002	0.541	0.460	0.081
cg01580782	5	927,438	927,437	non-genic	intergenic	0.002	0.529	0.470	0.059
cg03659519	18	74,961,967	74,961,966	*GALR1*	TSS200	0.005	0.570	0.430	0.141
cg26252794	14	88,097,222	88,097,221	non-genic	intergenic	0.006	0.526	0.474	0.053
cg01364862	3	159,364,475	159,364,474	*IQCJ-SCHIP1*	Body	0.006	0.470	0.530	−0.060
cg14051544	3	170,303,287	170,303,286	*CLDN11*	5′UTR	0.007	0.556	0.447	0.109
cg00363813	12	6,664,873	6,664,872	*IFFO1*	1st exon	0.016	0.554	0.446	0.108
cg10094616	8	53,478,024	53,478,023	*ALKAL1*	TSS200	0.018	0.543	0.456	0.087
cg14884793	13	109,148,554	109,148,553	non-genic	Intergenic	0.019	0.534	0.466	0.068
cg00082235	12	6,664,537	6,664,536	*IFFO1*	1st exon	0.032	0.538	0.462	0.076

Chr: chromosome; NR: non-recurrence; R: recurrence; UTR: untranslated region; TSS: transcription start site.

## Data Availability

The data presented in this study are available on request from the corresponding author. The data are not publicly available due to a data sharing agreement being processed among the collaborators.

## Supplementary Materials

The following are available online at https://www.mdpi.com/article/10.3390/cancers13122915/s1, Figure S1: Barretos Cancer Hospital Biobank’s routine of storage and DNA isolation from cryopreserved samples, Figure S2: Sample quality control and linear regression model. (A) Quality control of the samples included in the methylation array. (B) QQ-plot of the selected regression model. Observed *p*-values (black dots) plotted against the expected *p*-values under no association (red line), Figure S3: Multidimensional scaling (MDS) plots based on DNAme of the 14-CpG marker panel showing sample clustering according to different clinical variables. Each dot represents one OSCC patient sample in the discovery set, Figure S4: Box-plots based on DNAme of the proposed 14-CpG marker panel, stratified according to different clinical variables, Figure S5: Influence of anatomical sites in DNAme patterns. (A) Multidimensional scaling (MDS) plot using the β-values of the identified 14-CpG marker panel in the discovery cohort. (B) Box-plot using the β-values of the common 7 CpGs from TCGA tumor samples (*n* = 144; Alveolar Ridge = 14; Floor of Mouth = 35 and Oral Tongue = 95), Table S1: Differentially Methylated Positions (DMPs) between recurrent and non-recurrent OSCC patients, Table S2: Enriched pathways identified with gene set enrichment analysis using differentially methylated position (DMP)-related genes, Table S3: Differentially Methylated Regions (DMRs) between recurrent and non-recurrent OSCC patients.
