# Supplementary material for: DNA Methylation Markers from Negative Surgical Margins Can Predict Recurrence of Oral Squamous Cell Carcinoma
Source: Cancers (Basel). 2021 Jun 11;13(12):2915. doi: 10.3390/cancers13122915 (PMC8230600; doi:10.3390/cancers13122915)
Supplement: Supplementary file 1 [file cancers-13-02915-s001.zip › Figure S1.pptx]

## Slide 1
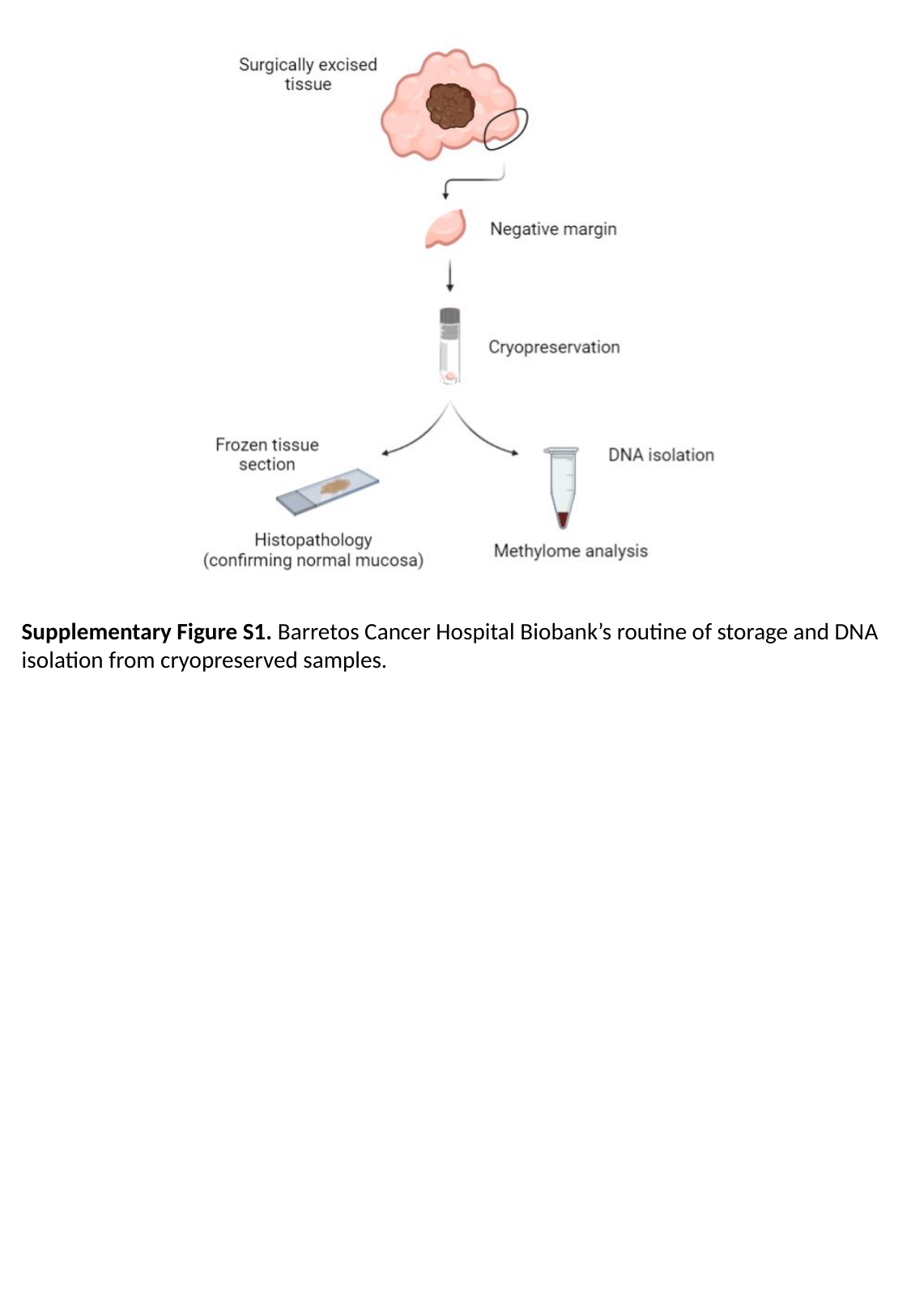

.
Supplementary Figure S1. Barretos Cancer Hospital Biobank’s routine of storage and DNA isolation from cryopreserved samples.
