# Supplementary material for: DNA Methylation Markers from Negative Surgical Margins Can Predict Recurrence of Oral Squamous Cell Carcinoma
Source: Cancers (Basel). 2021 Jun 11;13(12):2915. doi: 10.3390/cancers13122915 (PMC8230600; doi:10.3390/cancers13122915)
Supplement: Supplementary file 1 [file cancers-13-02915-s001.zip › Figure S2.pptx]

## Slide 1
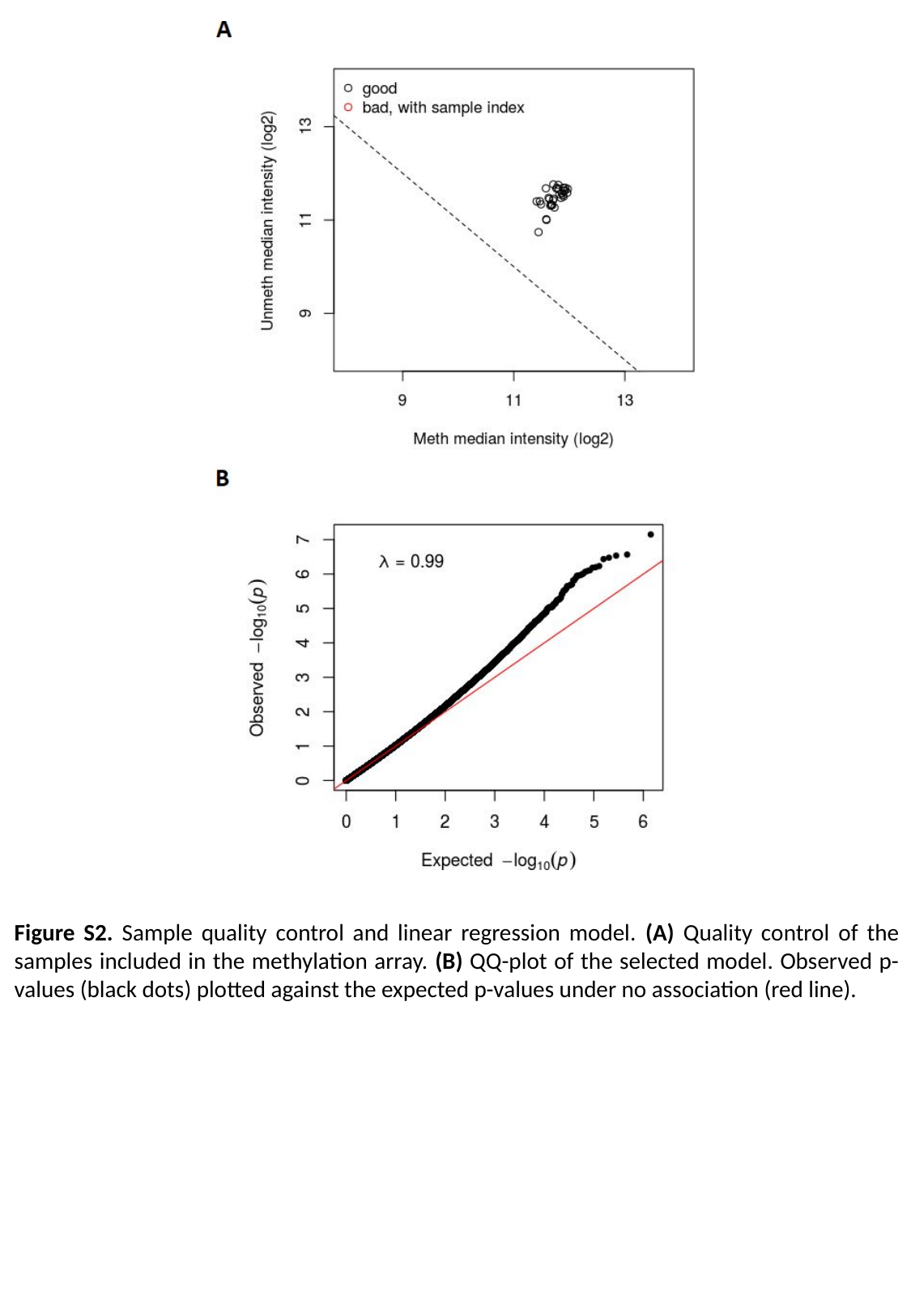

Figure S2. Sample quality control and linear regression model. (A) Quality control of the samples included in the methylation array. (B) QQ-plot of the selected model. Observed p-values (black dots) plotted against the expected p-values under no association (red line).
