# Supplementary material for: DNA Methylation Markers from Negative Surgical Margins Can Predict Recurrence of Oral Squamous Cell Carcinoma
Source: Cancers (Basel). 2021 Jun 11;13(12):2915. doi: 10.3390/cancers13122915 (PMC8230600; doi:10.3390/cancers13122915)
Supplement: Supplementary file 1 [file cancers-13-02915-s001.zip › Figure S3.pptx]

## Slide 1
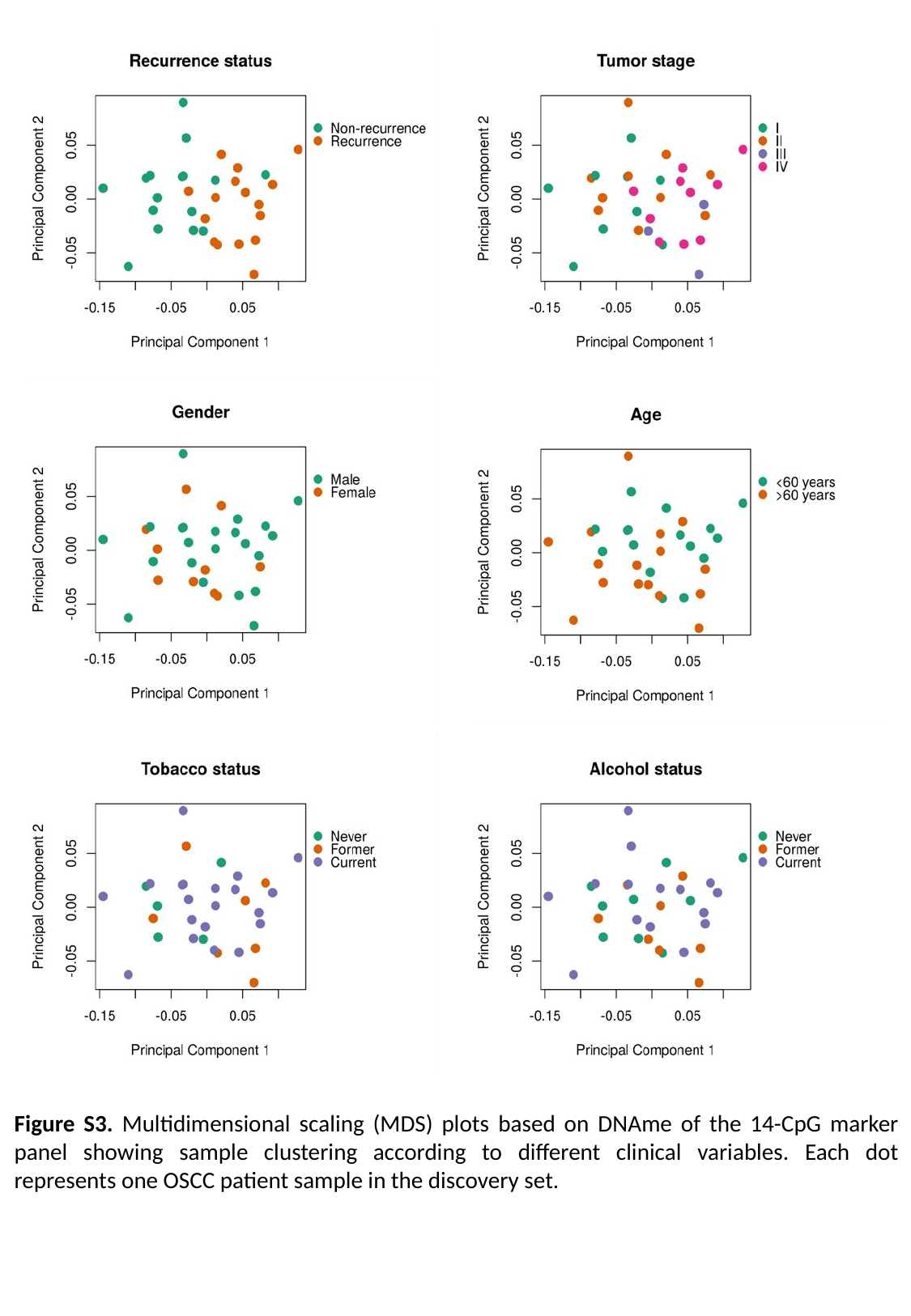

Figure S3. Multidimensional scaling (MDS) plots based on DNAme of the 14-CpG marker panel showing sample clustering according to different clinical variables. Each dot represents one OSCC patient sample in the discovery set.
