# Supplementary material for: DNA Methylation Markers from Negative Surgical Margins Can Predict Recurrence of Oral Squamous Cell Carcinoma
Source: Cancers (Basel). 2021 Jun 11;13(12):2915. doi: 10.3390/cancers13122915 (PMC8230600; doi:10.3390/cancers13122915)
Supplement: Supplementary file 1 [file cancers-13-02915-s001.zip › Figure S5.pptx]

## Slide 1
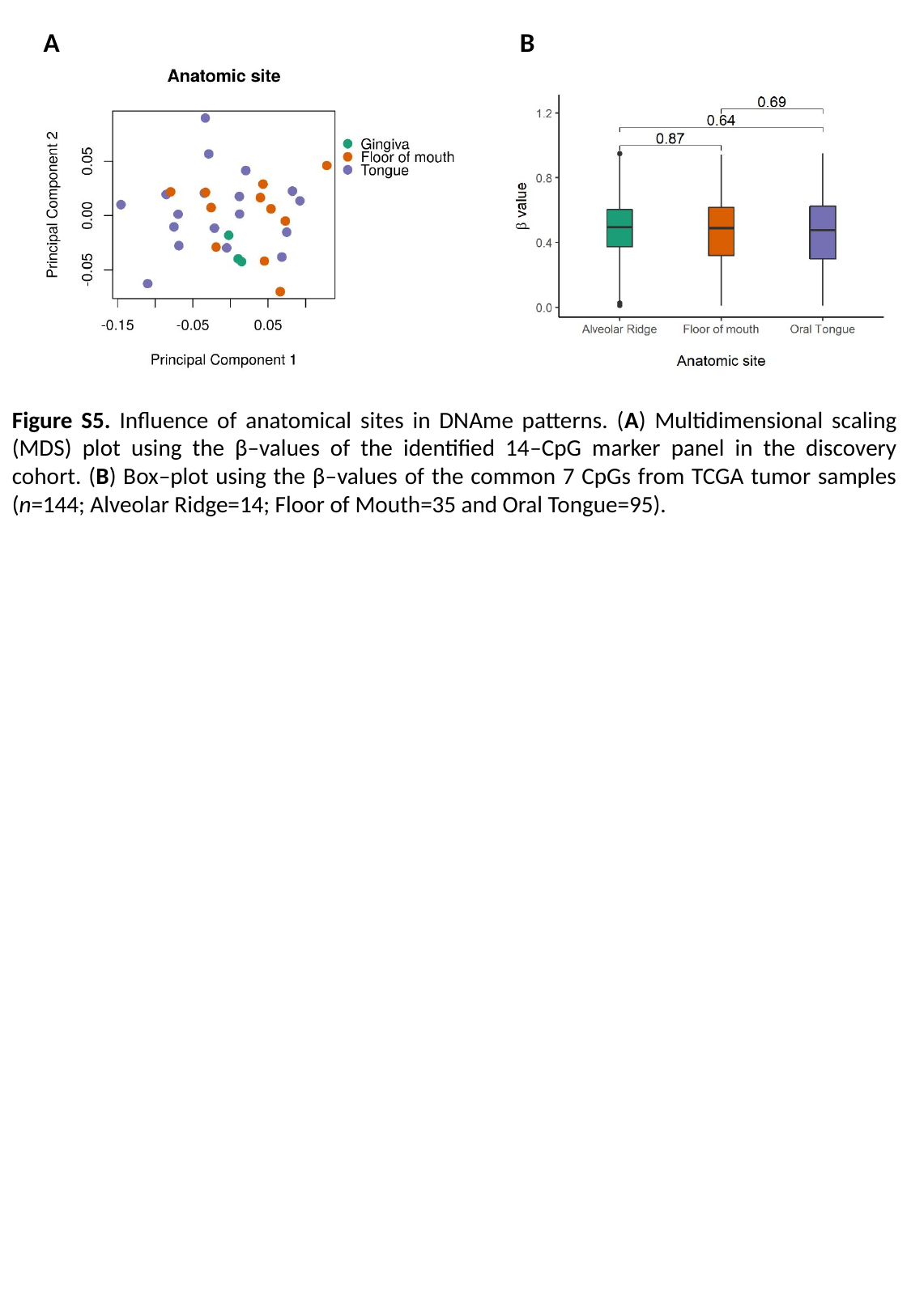

A
B
Figure S5. Influence of anatomical sites in DNAme patterns. (A) Multidimensional scaling (MDS) plot using the β–values of the identified 14–CpG marker panel in the discovery cohort. (B) Box–plot using the β–values of the common 7 CpGs from TCGA tumor samples (n=144; Alveolar Ridge=14; Floor of Mouth=35 and Oral Tongue=95).
